# Stakeholder Perceptions of Key Aspects of High-Quality Cancer Care to Assess with Patient Reported Outcome Measures: A Systematic Review

**DOI:** 10.3390/cancers13143628

**Published:** 2021-07-20

**Authors:** Angela M. Stover, Rachel Kurtzman, Jennifer Walker Bissram, Jennifer Jansen, Philip Carr, Thomas Atkinson, C. Tyler Ellis, Ashley T. Freeman, Kea Turner, Ethan M. Basch

**Affiliations:** 1 Department of Health Policy and Management, University of North Carolina, Chapel Hill, NC 27599, USA; stoveram@email.unc.edu; 2 Lineberger Comprehensive Cancer Center, Chapel Hill, NC 27599, USA; jansenj@email.unc.edu (J.J.); pmcarr@email.unc.edu (P.C.); 3 Department of Health Behavior, University of North Carolina, Chapel Hill, NC 27599, USA; kurtzmar@live.unc.edu; 4 Health Sciences Library, University of North Carolina, Chapel Hill, NC 27599, USA; jennifer_walker@unc.edu; 5 Memorial Sloan Kettering Cancer Center, New York, NY 10065, USA; atkinsot@mskcc.org; 6 Department of Surgery, University of Louisville Health, Louisville, KY 40202, USA; clayton.ellis@ulp.org; 7 British Columbia Cancer Agency, Vancouver, BC V5Z 1G1, Canada; ashley.freeman@bccancer.bc.ca; 8 Department of Health Outcomes and Behavior, Moffitt Cancer Center, Tampa, FL 33612, USA; kea.turner@moffitt.org; 9 Department of Medicine, University of North Carolina, Chapel Hill, NC 27599, USA; ebasch@med.unc.edu

**Keywords:** quality of healthcare, high-quality care, patient-centered care, cancer care delivery, patient reported outcome measures, symptoms, stakeholder perceptions, systematic review, quality of life

## Abstract

**Simple Summary:**

We conducted a review to identify important symptoms reported by patients on questionnaires (e.g., pain) that can be used to compare cancer centers on how well they provide care. For example, cancer centers could be compared on the percentage of patients with controlled pain after adjusting for demographic and clinical characteristics. Standard review methods were used to identify studies through August 2020. Searches generated 1813 articles and 1779 were coded as not relevant. The remaining 34 studies showed that patients, caregivers, clinicians, and healthcare administrators identify psychosocial care (e.g., distress) and symptom management as critical parts of high-quality care. Patients and caregivers also perceive that maintaining physical function and daily activities are important. Clinicians and healthcare administrators perceive control of specific symptoms to be important (e.g., pain, poor sleep, diarrhea). Results were used to inform testing of symptom questionnaires to compare the quality of care provided by six cancer centers.

**Abstract:**

Performance measurement is the process of collecting, analyzing, and reporting standardized measures of clinical performance that can be compared across practices to evaluate how well care was provided. We conducted a systematic review to identify stakeholder perceptions of key symptoms and health domains to test as patient-reported performance measures in oncology. Stakeholders included cancer patients, caregivers, clinicians, and healthcare administrators. Standard review methodology was used, consistent with PRISMA (Preferred Reporting Items for Systematic Reviews and Meta-Analyses). MEDLINE/PubMed, EMBASE, and the Cochrane Library were searched to identify relevant studies through August 2020. Four coders independently reviewed entries and conflicts were resolved by a fifth coder. Efficacy and effectiveness studies, and studies focused exclusively on patient experiences of care (e.g., communication skills of providers) were excluded. Searches generated 1813 articles and 1779 were coded as not relevant, leaving 34 international articles for extraction. Patients, caregivers, clinicians, and healthcare administrators prioritize psychosocial care (e.g., distress) and symptom management for patient-reported performance measures. Patients and caregivers also perceive that maintaining physical function and daily activities are critical. Clinicians and administrators perceive control of specific symptoms to be critical (gastrointestinal symptoms, pain, poor sleep). Results were used to inform testing at six US cancer centers.

## 1. Introduction

Performance measurement is the process of collecting, analyzing, and reporting standardized measures of clinical performance that can be compared across practices to evaluate how well care was provided [1]. In multi-payer systems like the United States (US) and some universal health systems like the United Kingdom (UK), performance measures are used for payment and quality improvement [2,3,4,5]. Braithwaite and colleagues [5] compared performance measures used in eight countries (Australia, Canada, Denmark, England, the Netherlands, New Zealand, Scotland, and the United States). More than 400 performance measures were identified, 45 of which were used in at least two countries. Most countries linked performance measures to a specific clinical condition, with the most common being safety, effectiveness, and access. There was a split in how countries ultimately used the performance measures, with some countries emphasizing public reporting and accountability (e.g., the UK National Health Service “star-ratings” system) and some countries using feedback to organizations to stimulate improvement [5]. 

Common performance measures in oncology rely on administrative data from electronic health records (e.g., 30-day readmission rates) or patients’ perceptions of their care experiences (e.g., communication skills of providers, ease of access to healthcare services) [2,3]. Rarely do performance measures include patient-reported symptoms or physical functioning, outcomes best captured with patient-reported outcome measures (PROMs). “PRO-Performance Measures (PRO-PMs)” is the standard terminology coined by the National Quality Forum [6] to denote using patients’ perspectives on how they feel and function as the performance measure [7,8,9]. For example, cancer centers could be compared on the percentage of patients with controlled pain after adjusting for demographic and clinical characteristics. 

PRO-PMs will become increasingly important for cancer centers to collect and track as the US and other countries move toward alternative payment models that emphasize patient-reported health outcomes as performance measures, such as the proposed Oncology Care First Model [10]. However, there is no consensus on what symptoms and health domains are most appropriate to assess for PRO-PMs in oncology [11,12]. Symptom management and quality of life are well documented as important to patients and health professionals alike [13,14,15], but these stakeholder groups may prioritize different health domains when considering quality of care. These nuances may be missed because studies commonly focus on the perspective of one stakeholder group. For example, studies have exclusively focused on the perceptions of oncology nurses [16,17], oncologists [18,19,20], or adults with cancer [21,22], and only rarely examine more than one stakeholder group at a time [13,14]. Stakeholder groups, such as healthcare administrators, are often excluded from studies in this area even though the use of PROMs and PRO-PMs in a clinic would necessitate their buy-in. In addition to a narrow focus on one stakeholder group, studies identifying perceptions of high-quality care may not make a distinction between countries with single-payer or universal healthcare systems vs. multi-payer systems. There may be cultural and payer differences in what symptoms and quality of life domains are prioritized based on the system [5]. 

To address these issues identified in prior studies, we made several enhancements in our systematic review. First, we expanded the stakeholder groups from cancer patients and clinicians to include hospital administrators or quality officers and caregivers. Second, we categorized extracted articles by stakeholder group to enable comparisons of symptom and health priorities for care. Third, we also categorized articles as in or outside the US to determine if there are geographic differences.

This systematic review is part of a larger research study to develop and test PRO-PMs for oncology [7] based on recommended best practices [6,9]. The goal is to develop adjusted PRO-PMs using existing PROMs that have validity and reliability evidence in cancer patients, rather than create new items [7]. However, there is no consensus for critical health areas to assess for PRO-PMs and which demographic and clinical characteristics will be important adjustment variables for oncology. Previously, we interviewed 124 stakeholders from six US cancer centers and national experts [7]. Stakeholder groups included patients and patient advocates, caregivers, nurses, oncologists, healthcare administrators, and national thought leaders. Interview participants prioritized the following list of symptoms to test as PRO-PMs for systemic therapy: gastrointestinal symptoms (diarrhea, constipation, nausea, vomiting); depression/anxiety; pain; insomnia; fatigue; dyspnea; physical function; neuropathy [7]. The current systematic review will help ascertain how generalizable these symptom priorities are for PRO-PMs in oncology. 

## 2. Results

**Figure 1:**
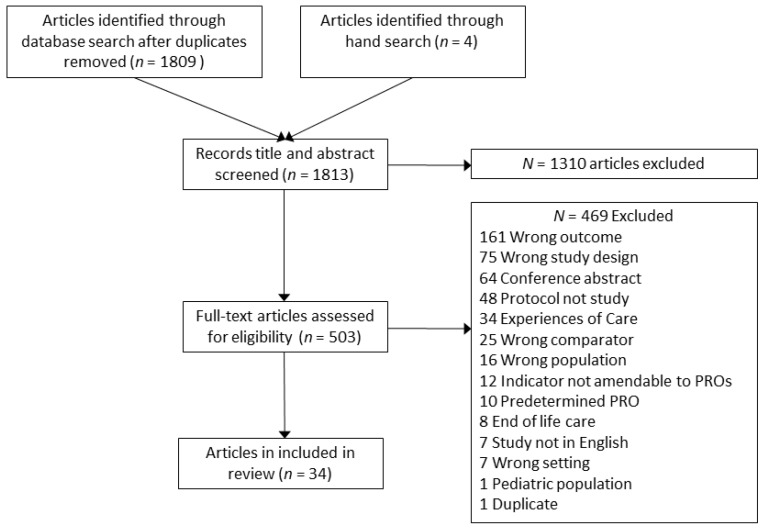
PRISMA diagram for article inclusion.

Systematic searches in MEDLINE/PubMed, EMBASE, and the Cochrane Library yielded 1809 unique articles, and hand-searching reference lists added 4 articles, for a total of 1813 articles. The majority, 1310 articles, were coded as irrelevant during the title and abstract review phases and another 469 were discarded during full-text review, leaving 34 articles for extraction. Figure 1 shows the PRISMA diagram. We excluded articles focusing exclusively on experiences of care, efficacy and effectiveness studies, end of life, newer therapies such as immunotherapy, and indicators not amenable to measurement with PROMs. 

Table 1 shows that patients were included in nearly all studies on stakeholder perceptions of quality of care, and 19/34 studies were restricted to patients only. Nine additional studies included patient perspectives along with providers and/or family members. Six studies focused on perspectives of leadership, policy experts, oncology social workers, nurses, or health services researchers. Across studies, the most common research design was interviews (14/34 studies), and interviews with questionnaires were used in five additional studies. Five studies used questionnaires exclusively and the remaining 10 studies used an alternative method or combination of methods. 

The majority of studies (20/34 (59%)) were conducted outside the US, mostly from Europe (12), Canada (2), Australia (2), the Middle East (2), or multiple countries (2). Fourteen studies (41%) were conducted in the US. In studies outside of the US, 12/20 (60%) studies examined multiple cancer types in the same study, while 3/14 (21%) studies in the US examined more than one cancer type.

Table 2 describes the 20 studies conducted outside of the US, including the location or setting, cancer type(s), treatment type(s), study design, stakeholder group(s), and sample size. The last column shows the important aspects of providing high-quality cancer care identified in each study that can be assessed with PROMs. Studies conducted outside of the US were published in 2005–2020. Table 3 describes the 14 studies conducted in the US between 2005 and 2020.

In the sections below, we descriptively examine patterns of extracted data in several ways, including study location (in or outside US), stakeholder group, and by broad categorizations of physical symptoms, psychosocial symptoms, and other symptoms. 

### 2.1. Study Location (in or Outside US)

**Figure 2:**
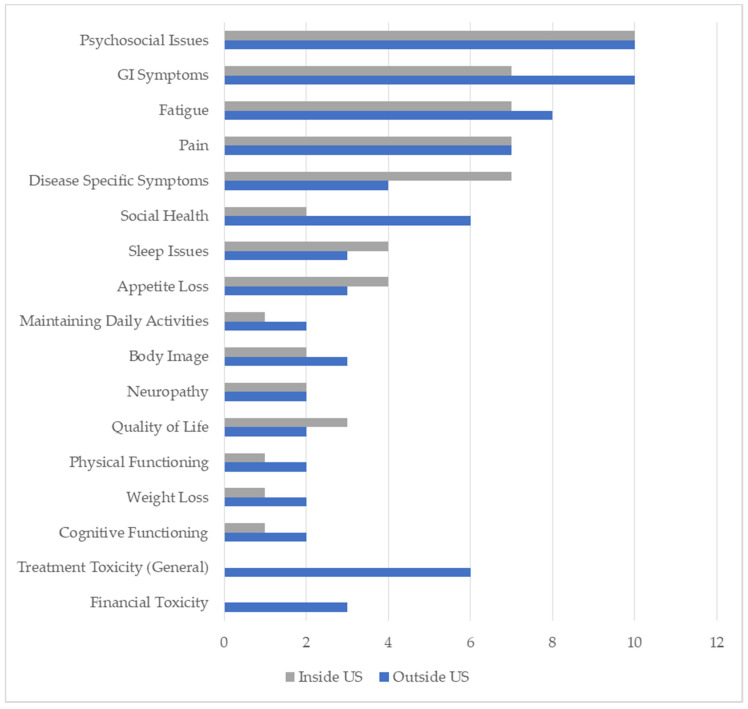
Number of articles per identified domain by location (in/outside US).

Figure 2 shows the number of articles identified for each symptom domain, separated by whether the study was conducted in or outside the US. There were no obvious patterns by study location. Both studies in and outside the US showed that the top four domains perceived to be important for high-quality cancer care were psychosocial symptoms (20/34 studies), gastrointestinal (17/34), pain (14/34), and fatigue (15/34). Three-quarters of the US studies (10/14 studies) identified psychosocial concerns as important for PRO-PMs. Symptoms specific to cancer types were the next most common (11/34), and less common outcomes included appetite loss, sleep issues, quality of life, financial toxicity, body image, cognitive issues, social health, physical function, and maintaining daily activities.

### 2.2. Symptom Domains and Stakeholder Groups

#### 2.2.1. Psychosocial Symptoms

##### Studies Exclusively with Patients

Patients with different cancer types, treatment types, and geographic location noted treatment for psychosocial concerns including depression, anxiety, and distress as being important for high-quality care delivery. In Sweden, Leo Swenne et al. conducted interviews with patients diagnosed with peritoneal carcinomatosis who discussed depression and worry following surgery [28]. In interviews with a sample of patients in Iceland, Hjorleifsdottir et al. found distress related to physical symptoms overwhelms patients and providing support for psychological problems was perceived to be important for high-quality care [27]. Gough et al. in the United Kingdom found that psychological domains impacting health-related quality of life included worry about symptoms and treatment scans [34]. A study focused on patients’ perspectives of nursing care also reported that management of psychological symptoms is important [25]. 

Four studies out of 14 from the US highlighted psychosocial concerns. In studies of women with breast cancer, distress was reported by most women regardless of treatment type and was one of the most common symptoms throughout treatment [47,50]. Williams et al. found that while physical symptoms were most concerning to patients with head and neck cancer and pelvis cancer, psychosocial concerns including anxiety and depression were more concerning to women with breast cancer [51]. 

##### Studies with Patients and Other Stakeholders

Three US studies with patients and clinicians focused on psychosocial concerns. In interviews with providers and patients with locally advanced or metastatic pancreatic cancer, detecting psychological concerns at diagnosis was perceived to be important for high-quality care. Clinicians noted that psychological and emotional symptoms tended to outlast physical symptoms [49]. In two studies of patients with prostate cancer and their clinicians, depression and anxiety were identified as key treatable concerns [42,52]. 

Three additional US studies did not include patients. In a symptom management clinic, Graze et al. discussed that high-quality nursing care delivery models include timely assessments for unmet needs and symptoms and effective symptom management for a variety of symptoms including distress and psychological symptoms [43]. Nelson et al. interviewed nurses, nursing assistants, and administrators who noted patients with high levels of anxiety need more support from staff to achieve high-quality care [17]. Aiello Bowles et al. interviewed policy experts, cancer care providers, and researchers who reported that treating psychosocial concerns was an important aspect of providing patient-centered care [45].

Four studies outside the US discussed treating psychosocial concerns during care. In a questionnaire and ranking study, clinicians identified insufficient attention to patients’ psychosocial distress as one of the top ranked causes of medication errors in cancer care [33]. In interviews, oncologists in Iran noted the importance of providing psychosocial care services to patients and families throughout treatment and the need for comprehensive support [20]. Kotronoulas et al. conducted interviews with nurses who reported patients need psychological and emotional support throughout treatment [36]. In a cross-sectional survey of members of the Association of Oncology Social Work, the majority of social workers reported patients’ fears, anxiety, depression, and distress were barriers to receiving optimal care [29]. 

Two studies also included family members as a stakeholder group. In Canada, Griffiths et al. conducted interviews with patients, family members, and nurses who reported treating psychosocial concerns was important during the transplant process [25]. Wagner et al. collected data from providers, patients, and family members who reported inadequate attention to emotional and social problems is a barrier to high-quality cancer care [46].

#### 2.2.2. Physical Symptoms

Physical symptoms were important to all stakeholder groups, but there was considerable variation in specific symptoms perceived to be critical for providing high-quality care.

##### Studies Exclusively with Patients

Outside the US, nine studies focused on patient perceptions of physical symptoms associated with high-quality cancer care across a variety of cancer types and treatments. In the UK, patients living with soft tissue sarcoma reported that important physical symptoms to treat include pain, fatigue, physical function, social functioning, and general side effects of treatment [34]. In Denmark, Holländer-Mieritz conducted interviews with patients with head and neck cancers who reported oral pain, decreased appetite, fatigue, and other disease-specific symptoms were important for high-quality care [35]. In Spain, Arraras et al. conducted interviews with patients who reported symptoms including impaired cognitive functioning, social functioning, fatigue, nausea, and vomiting were key symptoms [24]. In Iceland, patients with cancer noted physical symptoms, physical functioning, and distress [27]. Wang et al. conducted a mixed methods study in Ontario, Canada and found that significant and prevalent symptoms to treat include fatigue, decreased appetite, pain, nausea, and difficulty tasting [39]. In Jordan, Al-Jauissy et al. interviewed patients receiving chemotherapy who indicated that an important unmet need during their care was being dependent on others to maintain their daily activities [23].

Three studies outside the US were focused on patient perceptions of high-quality cancer care when receiving a specific therapy (two for chemotherapy and one for surgery). In Denmark, breast cancer patients receiving adjuvant chemotherapy reported important side effects to treat include gastrointestinal symptoms, neuropathy, sleep issues, cognitive issues, fatigue, and general cancer symptoms [32]. In Paris and Northern France, Sibeoni et al. conducted interviews with patients receiving chemotherapy who reported serious side effects, and impact on quality of life and ability to maintain daily activities were key treatment areas [37]. In Sweden, patients diagnosed with peritoneal carcinomatosis (thin layer of tissue covering most of the abdominal organs) reported that key symptoms were severe symptoms, including surgical site pain, poor appetite, and difficulty sleeping [28].

In the US, five studies focused on patient perceptions of physical symptoms associated with high-quality cancer care across a variety of cancer types and treatments. Three studies were with women with breast cancer. Chen et al. found largely disease-specific symptoms (arm swelling, arm pain) were important symptoms for women, but Thind and Whisenant reported a greater range of important symptoms to patients including pain, gastrointestinal symptoms, fatigue, and appetite loss [41,47,50]. Degboe et al. conducted interviews with patients diagnosed with metastatic or recurrent head and neck cancer who reported pain, fatigue, and other disease-specific symptoms including difficulty swallowing and slurred speech were important areas for treatment [48]. Williams et al. asked a diverse sample of patients with cancer to rank their most severe symptoms. Patients’ top-ranked symptoms included burning, painful, or dry skin, nausea, diarrhea, loss of appetite, and fatigue [51]. 

##### Studies with Patients and Other Stakeholders

Four studies included both patients and healthcare professionals in their samples. In a survey in the UK focused on chemotherapy/radiotherapy, Vidall et al. found that half of patients had experienced nausea severe enough to disrupt their daily activities. Healthcare professionals had tended to overestimate the incidence of nausea and vomiting but underestimated the severity and impact on patients’ lives [38]. Herman et al. conducted a study in the US with patients with pancreatic cancer and their providers and noted a wide range of symptoms impairing physical functioning were important to stakeholders [49]. Eton et al. conducted interviews with patients with metastatic hormone-refractory prostate cancer and providers who reported 11 concerns were relevant and important to providing high-value care [42]. These symptoms were similarly noted as important by physicians who treat patients with prostate cancer who perceived the most important signs and symptoms to patients are fatigue, bone pain, stress, anxiety, depression, and interference with daily activities [52].

Two studies outside the US examined key physical symptoms. At an interdisciplinary consensus meeting in Europe focused on therapeutic management in gynecological oncology, patients and physicians agreed on the importance of treating side effects from chemotherapy in evaluating cancer care. The most concerning side effects discussed were neuropathy, nausea, vomiting, and fatigue [31]. Salarvand interviewed oncologists in Iran who noted the most important part of providing high-quality cancer care is to manage all chemotherapy side effects and toxicities, but they did not specify specific symptoms to treat [20]. 

#### 2.2.3. Symptom Control

Effective symptom management was mentioned in multiple studies as an important component of high-quality cancer care. In Jordan, Al-Jauissy interviewed patients receiving chemotherapy who identified existing needs and that 50% of needs were not being met by care teams, particularly pain management and managing side effects [23]. In Norway, Kvale et al. interviewed patients on an oncology ward who reported symptom relief, and pain and nausea management, were important for providing high-quality care from nurses [22]. In England, Griffiths et al. surveyed patient with cancer about their perceptions of quality of care, emotional support, and support for symptom management [40]. Additionally in the UK, Gough et al. found symptom control for soft tissue sarcoma patients was important to maintaining quality of life [34]. In a study investigating symptom management of patients with lung cancer from rural and urban clinics in Western Australia, Hall et al. found differences in symptoms reported by setting, where metro/urban patients perceived pain management to be part of high-quality care but less so in rural areas [26]. Additionally in Australia, Wainer et al. discussed poor pain management for patients with gynecological cancers as low-quality care [30]. In interviews and surveys with patients with diverse cancer types, Schulmeister noted the need for symptom management and supportive care services in the provision of high-quality care [44]. Graze et al. developed a clinic led by advanced oncology nurses that specifically addressed symptom management to provide better care, and to reduce hospitalizations [43]. 

#### 2.2.4. Additional Domains

Less common outcomes included quality of life, financial toxicity, body image, spiritual health, and social health, although their use as PRO-PMs is debated because clinicians and health systems may not be able to influence these outcomes for quality of care [3,7,9,11]. Financial toxicity was reported in three studies related to provision of high-quality care. In Jordan, patients perceived help with financial toxicity to be an important part of their cancer care [23]. In a survey of oncology social workers, 49% of respondents listed inability to pay for treatment-related expenses as a major barrier to high-quality care [29]. Oncologists in Iran noted the importance of providing financial support and discussed the high costs of cancer treatment, and the financial burden patients face [20]. 

Maintaining quality of life was also noted in several studies as a key indicator of care quality, although studies rarely defined the term quality of life. Some studies appeared to operationalize quality of life as symptoms, even though quality of life is typically thought of as a patient’s perception of a combination of their physical, mental, and social well-being [53]. For example, in the US, Islam et al. interviewed patients with advanced non-small cell lung cancer to ask how they define treatment success before and after chemotherapy. Patients’ definitions of treatment success changed after treatment, often to include maintaining “quality of life” and daily activities [21]. In a study of patients receiving autologous stem cell transplantation, Schulmeister noted measuring “quality of life” is important information on treatment experiences and providing treatment for adverse effects [44]. In their article discussing results of an interdisciplinary round table on PROMs and quality of care for gynecological cancers, Armbrust recommended that “quality of life” should be included as an outcome in clinical trials [31]. Aiello Bowles et al. interviewed 23 policy experts, cancer care providers, and researchers who noted “quality of life” performance measures can aid in achieving patient-centered care [45]. 

## 3. Discussion

Thirty-four articles examined stakeholder perceptions of how to measure high-quality cancer care with PROMs. Approximately half of the articles were published in 2005–2016 and half in 2016–2020, showing growth and interest over time (there were no restrictions by publication year). The majority of studies were cross-sectional interviews. 

The patient perspective was represented in nearly all studies, and clinicians were represented in about half. Our review included more types of stakeholders than two previous reviews [13,14]. The reviews by Hess and colleagues [14] and Colosia and colleagues [13] examined perceptions of clinicians, patients, and thought leaders, but are about a decade old. We expanded the stakeholder groups to include healthcare administrators, quality officers, and caregivers.

Across studies, the top four domains perceived to be important for high-quality cancer care were psychosocial symptoms, gastrointestinal symptoms, pain, and fatigue, which reflect prevalent symptoms during cancer treatment [54,55,56]. Other important symptoms included dyspnea, poor sleep, neuropathy, physical function, and maintaining daily activities. This systematic review result is consistent with our prior interview study [7] with patients with cancer, caregivers, clinicians, healthcare administrators, and national experts. It is also consistent with a discrete choice experiment with >2200 US adults that found the most valued domains to maintain during a chronic health condition were physical functioning, maintaining daily activities, and little to no pain [15].

We descriptively looked at patterns in the articles in several ways. We looked at patterns in studies conducted in or outside the US but did not see obvious differences in symptom priorities for performance measurement. We also compared stakeholder groups’ prioritized symptoms for PRO-PMs. Patients and caregivers prioritized symptom management, psychosocial care (depression, anxiety, distress), and maintaining physical function and daily activities. Clinicians and healthcare administrators prioritized patient psychosocial care (e.g., stress, anxiety) and controlling specific symptoms (nausea, constipation, diarrhea, pain, and poor sleep). 

Many of these aspects of high-quality cancer care are generalizable across cancer and treatment types, and thus it may be possible to identify a universal core set of PRO-PMs in oncology that could be supplemented with additional items for cancer treatment subgroups. Additionally, a universal core set of PRO-PMs could be evaluated for use in other chronic health conditions. Psychosocial needs, pain management, management of certain symptoms (e.g., poor sleep) and maintaining physical function and daily activities occur in many health conditions and could be examined as cross-cutting PRO-PMs [57]. Patient distress in particular has been shown to be a major driver of care in many chronic health conditions [58]. Symptoms that are more specific to cancer and its treatments (e.g., body image after cancer treatment) may be more limited for use with other chronic health conditions.

Several additional outcomes mentioned by stakeholder groups (quality of life, financial toxicity, body image, and social health) may not be ideal for use as PRO-PMs because clinicians and cancer centers may not be able to act on these outcomes [3,7,9,11]. A key attribute of PRO-PMs is that they are health domains that can be acted on or treated by clinicians [6]. For example, quality of life was identified in review articles, but it typically reflects a patient’s perception of a combination of their physical, mental, and social well-being, and thus may be less interpretable and treatable than individual symptoms during clinical care [53]. However, several articles appeared to use the term “quality of life” when discussing specific symptoms. Standardizing terms and providing definitions in future articles would help make sense of studies recommending quality of life assessments be used as PRO-PMs during treatment. Additionally, some of these additional outcomes (e.g., financial toxicity) could be tested as potential adjustment variables given their association with demographic and clinical characteristics. 

### 3.1. Future Directions

Psychosocial care (e.g., depression and distress), symptom control of common cancer symptoms, and maintaining physical function and daily activities were perceived to be important for providing high-quality cancer care across the studies included in this review. Future work is needed to map these stakeholder perceptions to existing standardized collections of outcomes recommended for oncology clinical trials called “core outcomes sets” [59]. A review found that only 16% of core outcome sets had patient input [60], and thus we anticipate that the stakeholder perceptions generated by this systematic review may be broader than those found in core outcome sets. For example, Reeve and colleagues [61] identified a core outcome set to assess in adult oncology clinical trials that included many of the symptoms identified in this review as important for routine care (fatigue, insomnia, pain, appetite loss, dyspnea, cognitive problems, anxiety, depression, neuropathy, nausea, constipation, and diarrhea). However, our review shows that cancer patients and caregivers also identify maintaining physical function and daily activities as critical aspects of providing high-quality care in routine cancer care, but these are absent in [61].

This systematic review is part of a larger study to develop and test PRO-PMs for oncology using existing PROMs rather than writing new items [7]. We combined the current review results with our prior interview data with >120 stakeholders [7] via expert consensus. The combined results informed testing of PRO-PMs at six US cancer centers [7]. The list of symptoms being tested as PRO-PMs included pain, gastrointestinal symptoms (nausea, vomiting, constipation, diarrhea), psychosocial symptoms (depression, anxiety), sleep issues and fatigue, neuropathy, appetite loss, physical function and daily activities, and shortness of breath [7]. Financial toxicity is being tested as a potential risk adjustment variable [7] since many cancer centers do not have services and resources related to it. Other potential risk adjustment variables being tested are insurance status, cancer type, age, race/ethnicity, and sex. 

PROMs selected to assess the key health domains are described in [7], as well as PRO-PM measure specifications, and patient feasibility and acceptability testing [7]. Briefly, 607/653 patients from six cancer centers completed the PROM items (93%) in wave 1 testing, which exceeded our a priori feasibility definition of 75% [7]. Most (>95%) participants found the PROM questions to be easy to understand and complete [7]. Analyses are in progress to aggregate individual PROM items to the cancer-center level and to empirically determine risk adjustment variables. PRO-PM measure specifications are being tested for each symptom individually (e.g., proportion of patients at a practice with well-controlled symptom (e.g., pain), as well as for multi-symptom summary measures. Quantitative analyses of PRO-PMs and risk adjustment variables will be reported elsewhere. This work is one of the necessary first steps to develop PRO-PMs for oncology, and more research will be needed to develop and test optimal PRO-PMs and adjustment variables across cancer types, stages, and treatment types.

### 3.2. Limitations

This review has several limitations. Our review identified stakeholder perceptions of how to measure high-quality cancer care. It is unknown whether the identified symptom priorities for PRO-PMs will generalize across cancer types, disease stages, treatment types, or even other chronic health conditions. Future research will be needed to empirically test whether a universal set of PRO-PMs can be developed for oncology, and the types of supplemental items needed for different cancer types and treatments. In this review, we excluded articles focused on newer therapies, such as immunotherapy, because they are associated with a different symptom profile and timing of symptoms. As this field develops, a specific review for immunotherapy PRO-PMs will be needed. 

Stakeholder engagement should continue to be an important part of PRO-PM development, testing, and implementation in oncology, with particular emphasis on patients and caregiver input. Future research should consider additional stakeholder groups that would enhance the PRO-PM development process, such as local and national payer organizations, palliative care teams, and supportive care services providers such as mental health providers.

Another limitation is that we excluded articles focusing exclusively on patient perceptions of care experiences (e.g., communication skills of providers, ease of access to healthcare services) because many patient reported experience measure (PREM) performance measures already exist and are commonly used for performance measurement in oncology. For example, the Consumer Assessment of Healthcare Providers and Systems (CAHPS^®^) is a PREM that is commonly used in the US as a performance measure [62,63,64]. However, it may be the case that perceptions of symptom control interact with experiences of care when stakeholders are considering the quality of the care delivered. Black et al. [65] examined whether patients receiving elective surgery may conflate outcomes (how they feel and function) with their care experiences. PROMs and PREMs were only weakly correlated (*r* = 0.2), suggesting PROMs and PREMs measure different aspects of care delivery and patients can and do distinguish these domains of quality [65]. Therefore, a combination of PROM and PREM performance measures may be needed to capture cancer patients’ and other stakeholders’ perceptions of the quality of care delivered. 

If both PROMs and PREMs are used as performance measures for oncology, more integrated administration and use by care teams will likely be needed. PROMs are usually administered before or during visits to inform the conversation on symptom detection and management between patients and clinicians [66]. PREMs are typically administered weeks to months after a visit in the US, sometimes by third party systems, and there may be limited feedback to clinics. Research is needed on optimal timing, administration, and implementation strategies for PROMs and PREMs to be used together as performance measures [66].

Simply adding more performance measures to already taxed healthcare systems is unlikely to improve care though. Existing quality of care frameworks already have hundreds of performance measures, and thus careful consideration of which PROMs and PREMs to add and retiring outdated measures will be needed [67]. Braithwaite and colleagues [5] found more than 400 performance measures when comparing frameworks across eight countries (Australia, Canada, Denmark, England, the Netherlands, New Zealand, Scotland, and the United States). One possibility is retiring outdated performance measures where clinicians must document that an action was completed to bill for that service in fee-for-service models [67]. For example, an existing US performance measure is documenting that a patient was screened for depression using a standardized tool [68], rather than tracking the depression scores over time to determine improvement in symptoms. The majority of US performance measures are process-oriented (~60%), and fewer than 5% use patient- or clinician-reported health status [69]. PRO-PMs could be major drivers of improvement in cancer care as the US and other countries move toward alternative payment models that emphasize both patient-reported health outcomes and care experiences.

## 4. Materials and Methods

### 4.1. Literature Search Strategy

**Figure 3:**
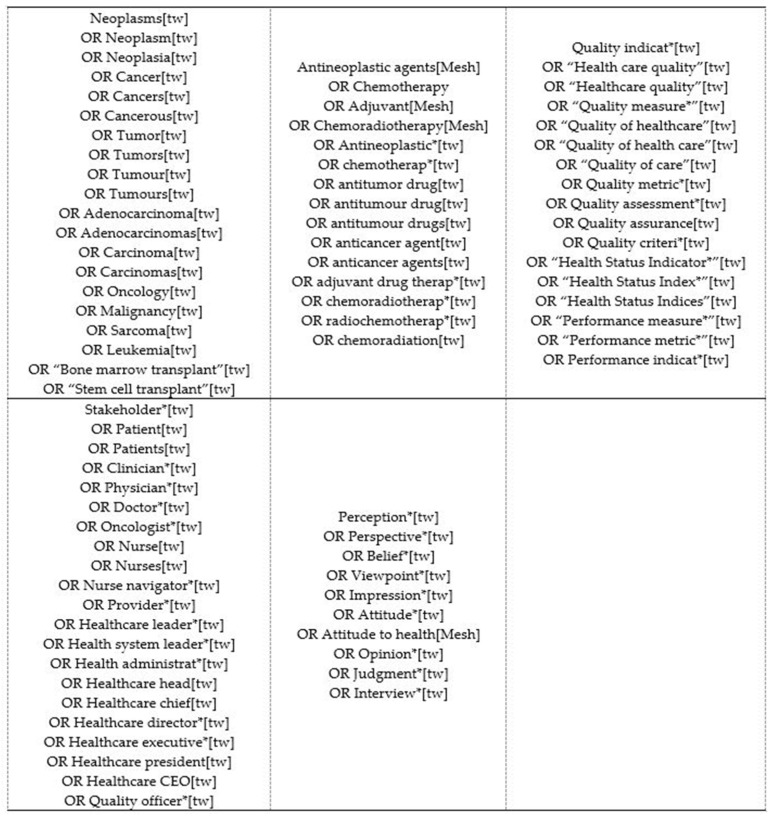
Search terms.

This study was exempt from oversight by the University of North Carolina at Chapel Hill IRB because there was no patient contact. Standard systematic review methodology was used, consistent with National Academy of Medicine standards and PRISMA (Preferred Reporting Items for Systematic Reviews and Meta-Analyses) reporting criteria [70,71,72]. Systematic searches were conducted by a health sciences librarian who has expertise in cancer systematic reviews. Steps included deciding which databases to search (MEDLINE/PubMed, EMBASE, and the Cochrane Library), identifying search strings appropriate for each database, and importing titles and abstracts into a reference manager for coding. The literature search included Medical Subject Headings (MeSH) and Emtree headings and related text and keyword searches when appropriate. Search strings were generated by the health science librarian and reviewed by the research team (including patient investigators) and scientific advisory board for potential additions. Figure 3 shows the search strings.

### 4.2. Inclusion and Exclusion Criteria

Studies were eligible for inclusion if they involved a key stakeholder group’s perception of what constitutes high-quality care for cancer, including symptoms, toxicities, adverse events, physical function, and other similar topics that can be reported by patients. Stakeholders included cancer patients, clinicians, healthcare leaders, quality officers, and caregivers. We excluded studies that used pediatric or adolescent populations, examined efficacy of treatment regimens, comparative effectiveness studies, PROM studies that did not mention stakeholder perceptions of high-quality care or key symptoms, studies focusing exclusively on patient experiences of care (e.g., communication skills of providers, ease of access to healthcare services), studies focused on survivorship (post-treatment), core outcome sets for clinical trials, newer treatments such as immunotherapy, and conference abstracts. Searches were limited to English-language studies or those with English translations available. No restrictions were placed on publication year. Search dates were from the databases’ inceptions to August 2020.

### 4.3. Coder Training

Four coders participated in training where 20 titles and abstracts were coded with a senior member of the research team. Inter-rater reliability was calculated with Krippendorf’s alpha in SAS with the KALPHA macro (distribution obtained with bootstrapping) [73]. Krippendorf’s alpha is recommended over kappa in cases where the codes are nominal (e.g., retain or reject abstracts) and there are more than two coders [73]. Krippendorf’s alpha scores exceeded the minimum threshold of 0.70 (Krippendorf alpha = 0.72, 95% CI: 0.59–0.84) [74]. In other words, coders agreed nearly three-quarters of the time whether an abstract should be retained or rejected. Discrepancies were discussed and resolved by consensus. 

### 4.4. Abstract Coding

Abstract coding was then done in stages by four coders with Covidence software (Melbourne, Australia). Two coders independently reviewed each entry (randomly assigned by the system) and a fifth coder resolved conflicts. Article titles were coded independently based on predetermined criteria (e.g., topic is related to care quality and delivery in cancer and could be reported by patients). Titles coded as “not relevant” by both coders were excluded and other titles retained. For retained titles, abstracts were then double-coded. Retained abstracts underwent a full-text review. Articles with disagreements were coded by a fifth coder (senior member of research team), until retained articles were all coded as relevant. Full-text articles were screened by two coders against the eligibility criteria. 

### 4.5. Extraction of Article Information

Articles meeting inclusion criteria had descriptive information extracted, including whether respondents were from the US or another country, cancer type(s), treatment type(s), study design, stakeholder group(s) assessed, and sample size. Each article was also reviewed by two coders for the symptoms, health, and quality of life domains perceived to be important for providing high-quality cancer care.

## 5. Conclusions

This systematic review showed that there are key patient-reported symptoms and health domains stakeholders perceive to be important for assessing the quality of cancer care delivered. Patients, caregivers, clinicians, and healthcare administrators prioritize psychosocial care (depression, anxiety, distress) and symptom management for patient-reported performance measures. Patients and caregivers also perceive that maintaining physical function and daily activities are critical. Clinicians and healthcare administrators perceive control of specific symptoms to be critical (gastrointestinal symptoms, pain, and poor sleep). Results were used to inform testing of patient-reported performance measures at six US cancer centers.

## Figures and Tables

**Table 1:** Characteristics of 34 studies included in review.

Study Characteristics	Studies Conducted Outside of US (*n* = 20)	Studies Conducted in US (*n* = 14)
**Stakeholder Group**		
Patients	12	7
Patients + other group	5	4
Non-patients only	3	3
**Methodology**		
Interviews	7	7
Interviews + questionnaire	2	3
Questionnaire only	5	0
Other methodology	6	4
**Cancer Type**		
Breast	1	3
Prostate	0	2
Gynecological	2	0
Lung	1	1
Colorectal or anal	1	0
Head and neck	1	1
Pancreatic	0	1
Soft tissue sarcoma	1	0
Multiple cancer types	12	3
Other	1	3

**Table 2:** Studies conducted outside of the US (*n* = 20).

First Author, Year	Setting	Location	Cancer Type(s)	Treatment Types (s)	Study Type	Stakeholder Group(s)	Sample Size	Important Aspects of Providing High-Quality Care
Al-Jauissy et al., 2009 [23]	Chemotherapy clinic at major university	Irbid, Jordan	Colon Lung Breast Urologic Stomach Pancreas Cervix	Chemotherapy	Interview and questionnaire	Patients	62	Pain management Side effects of treatment Able to do daily activities Financial toxicity Social health
Arraras et al., 2013 [24]	Day hospital	Pamplona, Spain	Multiple (not specified)	Chemotherapy with or without radiation or surgery	Questionnaire	Patients	176	Cognitive function Social health Fatigue Nausea/vomiting Financial toxicity
Griffiths, 2005 [25]	Tertiary care center	Ontario, Canada	Lymphoma Leukemia Breast	Chemotherapy	Observation + interviews	Patients, family, nurses	Patients: 11 Family: 5 Nurses: 8	Psychosocial/spiritual Side effects Toxicity
Hall et al., 2008 [26]	Rural and metropolitan clinics	Western Australia	Lung	Surgery, radiation, chemotherapy	Interviews and chart reviews	Patients and general practitioners	Patients: 43	Pain management
Kvale, et al., 2010 [22]	Regional hospital	Norway	Multiple (not specified)	Chemotherapy	Interviews	Patients	20	Pain management Nausea management Side effects Symptom relief Candida of mouth
Hjorleifsdottir and Hallberg, 2008 [27]	Outpatient oncology clinics	Iceland	Breast (44%) Prostate (12%) Ovarian (8%) Colon (8%) Other (28%)	Chemotherapy or radiotherapy	Interviews	Patients	25	Distress Physical symptoms Psychological well-being
Leo Swenne, et al., 2015 [28]	Surgical ward	Sweden	Peritoneal carcinomatosis	Surgery and chemotherapy	Interviews	Patients	19	Surgical site pain Poor appetite Difficulty sleeping Physical functioning
Burg et al., 2010 [29]	Not applicable	International	Not applicable	Not applicable	Questionnaire	Social workers	622	Patient fears, anxiety Depression and distress
Wainer et al., 2012 [30]	Cancer centers	Australia	Gynecological	Surgery, chemotherapy, radiation	Interview	Patients	25	Poor pain management
Armbrust, 2020 [31]	N/A	Europe	Gynecological	Chemotherapy “maintenance”	Consensus meeting	Advocates, oncologists	Not reported	Maintenance therapy symptoms
Bæksted, 2019 [32]	Oncology departments	Denmark	Breast cancer	Adjuvant chemotherapy	Interviews + online	Patients	Trial: 191 in ePROM arm	Nausea Fatigue Dry mouth Aching joints Neuropathy Sleeping issues Cognitive issues
Car, 2017 [33]	Not reported	North West London, UK	Not applicable	Not applicable	Questionnaire	Oncologists, general practitioners, nurses, pharmacists	40	Psychological distress
Gough, 2019 [34]	Specialized clinic	UK	Soft tissue sarcoma	Chemotherapy	Questionnaire + interview	Patients	66	Social/psychological Physical symptoms Pain Sleep disturbance
Holländer-Mieritz, 2019 [35]	Hospital	Copenhagen, Denmark	Head and neck	Radiotherapy with or without chemotherapy	Interview	Patients	13	Oral pain Decreased appetite Dysphagia Dry mouth Fatigue Hoarseness
Kotronoulas, 2017 [36]	Hospitals	Scotland	Colorectal cancer	Adjuvant chemotherapy	Literature review, focus groups, and questionnaire	Colorectal cancer nurses and patients	Focus groups: 8 patients, 7 nursesQuestionnaire: 14 patients	Psychological and emotional Social health Practical help (e.g., finances, work or child support)
Salarvand, 2017 [20]	Not reported	Iran	Multiple cancer types—non-metastatic (not specified)	Not applicable	Interview	Oncologists	15	Social support Treatment support Social health Psychological support Financial support
Sibeoni, 2018 [37]	Oncology departments	Paris and Northern France	Breast Lung Melanoma Lymphoma Urologic Prostate Ovary	Chemotherapy (oral or IV); chemotherapy + other treatment	Interview	Patients	30	Side effects Quality of life Maintain daily activities
Vidall, 2016 [38]	Not reported	United Kingdom	Any stage/any type cancer (not specified)	Chemotherapy/radiotherapy and received antiemetic	Questionnaire	Patients, nurses, and physicians	Patients: 78 Physicians: 75 Nurses: 31	Nausea Impact on daily life Symptom control
Wang, 2018 [39]	Cancer center	Toronto, ON, Canada	Gastrointestinal Genitourinary Breast Head and neck Hematologic	Chemotherapy	Medical record abstraction + questionnaire + interview	Patients	497	Fatigue Decreased appetite Pain Nausea Difficultly tasting
Griffiths, 2013 [40]	Hospital	United Kingdom	Multiple (not specified)	Surgery, radiation, and chemotherapy	Questionnaire	Patients	67,713	Emotional Symptom control

**Table 3:** Studies conducted in the US (*n* = 14).

First Author, Year	Setting	US Location	Cancer Type(s)	Treatment Type(s)	Study Type	Stakeholder Group(s)	Sample Size	Important Aspects of Providing High-Quality Care
Chen et al., 2008 [41]	103 hospitals	Los Angeles, CA	Stage I and II breast cancer	Chemotherapy, surgery, or hormone therapy	Interview + questionnaire	Patients	495	Arm swelling Arm pain Difficulty with arm movement
Eton et al., 2010 [42]	Not reported	Evanston, IL	Metastatic hormone-refractory prostate cancer	Chemotherapy, hormonal therapy, and bisphosphonate therapy	Literature review, patient interviews, practitioner surveys	Patients and providers	Patients: 45 Practitioners: 10	General pain, bone pain Genito-urinary symptoms Fatigue Appetite loss Constipation, diarrhea Peripheral neuropathy
Graze, et al., 2014 [43]	Cancer institute	Annapolis, MD	Multiple (not specified)	Not applicable	Literature review, interviews	Leadership and nursing staff	Not applicable	Fatigue Sleep Distress Dyspnea Pain Weakness Nausea, vomiting Diarrhea
Schulmeister, Quiett, and Mayer, 2005 [44]	Outpatient clinic	US	Breast: 59% Lymphoma: 19% Multiple myeloma: 14% Other: 9%	Chemotherapy	Interview + questionnaire	Patients	36	Long-term effects of treatment Symptom management Supportive care services
Aiello et al. 2008 [45]	Not applicable	US	Not applicable	Not applicable	Interviews	Policy experts, providers, researchers	Policy: 6 Providers: 6 Researchers: 13	Quality of life Emotional support
Nelson. 2011 [17]	Comprehensive cancer center	Southwestern US	Not applicable	Not applicable	Interviews	Nurses, nursing assistants, directors	RN: 10 Nursing Assistant: 5 Director: 5	Psychosocial issues
Wagner et al., 2010 [46]	Cancer centers, primary care providers, local patient advocacy	Washington, Massachusetts, and Michigan	Multiple (not specified)	“Finished with initial cancer treatment”	Interviews, focus groups and site visits	Patients, providers and family members	Interviews: 23 Focus groups: Providers: 15 Family: 18 Patients: 21	Inadequate emotional support Psychosocial issues
Thind, Hoq, Diamant, and Maly, 2010 [47]	Cancer treatment program	California	Breast cancer	Surgery, chemotherapy, or radiotherapy	Questionnaire + interviews	Patients	924	Pain Nausea Sadness
Degboe, 2018 [48]	Specialist clinical sites	Massachusetts and Tennessee	Recurrent/metastatic HNSCC	Chemotherapy and radiation—43%Radiation, chemotherapy, surgery—50%	Interview	Patients	14	QLQ-C30 and QLQ-H&N35 lack questions on excess mucus production and neuropathy Most significant impact: difficulty speaking, swallowing, pain, fatigue
Herman, 2019 [49]	Hospital	Patients: US; Providers: US and Europe	Pancreatic cancer	Surgery, radiation, chemotherapy, and immunotherapy	Interviews	Patients and clinicians	Patients: 24 Providers: 6	Pain: abdominal, back, upper GI Bowel/digestive problems, nausea Reduced appetite, weight loss Cognitive difficulties Hair loss Neuropathy Emotional/psychological/social Physical function
Islam, 2019 [21]	Cancer center	Midwestern States, Florida	Advanced non-small cell lung cancer	Chemotherapy	Interview	Patients	235	Quality of life Able to reach important personal goals/do routine activities
Whisenant, 2019 [50]	Cancer center	Texas	Breast	Chemotherapy +/- radiotherapy	Interview	Patients	36	36 symptoms Symptoms interfering with daily activities
Williams, 2020 [51]	Teaching hospital	Los Angeles, California	Breast, head and neck, pelvis	Radiation +/- other modalities	Card sorting/ranking	Patients	55	Physical side effects Psychosocial Affecting work/home duties
Holmstrom, 2019 [52]	Providers: academic institutions patients: recruited via social networking	US	Metastatic castration-resistant prostate cancer	Not applicable	Interview	Patients and physicians	Physicians: 3 Patients: 19	Urinary symptoms Bone pain, fatigue, hair loss Enlarged breasts Hot flush Muscle loss Inability to focus Stress, anxiety, depression Interference with daily activities
